# Effects of pamidronate disodium on the loss of osteoarthritic subchondral bone and the expression of cartilaginous and subchondral osteoprotegerin and RANKL in rabbits

**DOI:** 10.1186/1471-2474-15-370

**Published:** 2014-11-06

**Authors:** You Lv, Jie-yun Xia, Jing-yang Chen, Hui Zhao, Hai-cui Yan, Han-shi Yang, Qiang Li, Yu-xin Fan, Kai-jin Guo, Xiang-yang Chen

**Affiliations:** Department of Postgraduate, Xuzhou Medical University, 209 Tongshan Road, Xuzhou, 221004 Jiangsu, China; Department of Orthopedics, Affiliated Hospital of Xuzhou Medical University, 99 Huaihai Road, Xuzhou, 221002 Jiangsu China; Department of Orthopedics, Shanghai Changzheng Hospital, Shanghai, 200003 China

**Keywords:** Osteoarthritis, Pamidronate disodium, Subchondral bone, Cartilage, Osteoprotegerin (OPG), Receptor activator of nuclear factor-κ B ligand (RANKL)

## Abstract

**Background:**

Osteoarthritis (OA) is a major health problem in the increasingly elderly population. Therefore, it is crucial to prevent and treat OA at an early stage. The present study investigated whether pamidronate disodium (PAM), a bone-loss inhibitor, can significantly prevent or reverse the progression of early anterior cruciate ligament transection (ACLT)-induced OA. Whether therapeutic intervention is associated with regulation of the expression of osteoprotegerin (OPG), receptor activator of nuclear factor-κB ligand (RANKL), metalloproteinase-9 (MMP-9) or Toll-like receptor-4 (TLR-4) in cartilage and/or subchondral bone was also investigated.

**Methods:**

60 New Zealand rabbits were randomized into four groups: Sham-operated (*n* = 20); ACLT (*n* = 20); short-term treatment with PAM (PAM-S, *n* = 10) and long-term treatment with PAM (PAM-L, *n* = 10). For cartilage and subchondral bone testing, rabbits from Sham and ACLT groups were harvested at 2, 4, 6, and 14 weeks. Rabbits were given PAM from the 4th week after ACLT operation in PAM-S and PAM-L group, and were harvested at 6 and 14 weeks, respectively. Trabecular characteristics and cartilage changes were detected using Micro-CT, safranin O and rapid green staining, respectively. Immunohistochemical staining for OPG and RANKL were also performed. OPG, RANKL, MMP-9 and TLR-4 expression was evaluated by western blot analysis.

**Results:**

Micro-CT and histology analyses indicated that PAM treatment for 2 or 10 weeks could completely prevent or reverse osteoarthritic subchondral bone loss and cartilage surface erosion. Immunohistochemistry and western blot analysis indicated that expression of OPG and RANKL increased, although RANKL expression increased more significantly than that of OPG. Therefore the ratio of OPG to RANKL was lower in the ACLT group. However, the ratio of OPG to RANKL in the PAM group was significantly higher than that in the ACLT group. Additionally, expression of MMP-9 and TLR-4 were upregulated in the ACLT group and downregulated in the PAM treated groups.

**Conclusions:**

PAM can significantly inhibit and even reverse early osteoarthritic subchondral bone loss, thus alleviating the process of cartilaginous degeneration. The mechanisms involved may be associated with the upregulation of OPG expression, and downregulation of RANKL, MMP-9 and TLR-4 expression.

**Electronic supplementary material:**

The online version of this article (doi:10.1186/1471-2474-15-370) contains supplementary material, which is available to authorized users.

## Background

Osteoarthritis (OA) is a degenerative joint disease characterized by progressive cartilaginous degeneration, osteophyte formation, subchondral bone changes and synovial inflammation[1, 2]. Although cartilage lesions are the main feature of OA, increasing evidence indicates that it is also a bone disease[3, 4]. Radin et al.[5] were the first to propose that subchondral bone may cause cartilaginous degeneration. Thereafter, multiple reports have suggested that changes in subchondral bone may alter the load distribution, and so becoming an important inducing or accelerating cause for cartilaginous injury in OA[3, 6, 7]. Recent animal experiments have shown that early-stage OA is accompanied by a reduction in bone volume, indicating that the initial subchondral bone resorption may be a crucial factor for OA onset and progression[8, 9]. However, Bobinac et al.[10] have posited that changes in subchondral bone could exist during or after cartilaginous degeneration. Therefore, determining the exact effects of subchondral bone on the occurrence and development of OA, particularly during the early stage, is of crucial importance in seeking new diagnostic markers and therapeutic targets for OA[2, 4].

Subchondral bone mass is maintained through a balance between formation and resorption during the development of OA. In the bone resorption process, regardless of the pathology, the osteoclast is the exclusive resorptive cell[11]. A signaling molecule composed of osteoprotegerin/receptor activator of nuclear factor-κ B/RANK ligand (OPG/RANK/RANKL) has been described as critical in controlling osteoclast biology. RANKL, synthesized by osteoblasts, binds to RANK on the osteoclast precursor membrane and is an essential factor for osteoclast differentiation and bone resorption[12, 13]. OPG, which is also produced by osteoblasts, acts as a soluble decoy receptor for RANKL. Also, by interacting with RANKL, OPG prevents RANK activation and subsequent osteoclastogenesis, resulting in the inhibition of bone resorption[14]. It was recently reported that when OPG was transferred onto cartilage explants, it resulted in a marked decrease in aggrecan cleavage and cartilage proteoglycan release, demonstrating the role of OPG in the regulation of cartilage catabolism[1]. Thus, although the roles of these cytokines secreted by the chondrocytes in the onset and progression of subchondral bone changes in OA are hypothesized, they remain to be fully investigated.

A recent study has shown that cartilage damage in OA is caused by disruption of the balance between catabolic and anabolic function of chondrocytes[15]. Catabolic activities of OA chondrocytes are related to the elevated release of cartilage degrading enzymes, such as matrix metalloproteinases (MMPs)[16], while in anabolic activities, Toll-like receptor 4 (TLR4) has been shown to play a crucial role in inflammatory signaling in human chondrocytes[15].

As the goal of modern medicine shifts towards disease prevention, subchondral bone may represent an attractive candidate as a therapeutic target in early OA. Therefore, bone-loss inhibitors, such as pamidronate disodium (PAM) and other bisphosphonates, might be potential therapeutic drugs for OA[17]. Kadri et al. have proposed that the level of bone resorption influences cartilage metabolism and the inhibition of bone resorption might prevent the progression of OA[18]. In studies of ovariectomized mice, PAM was efficacious in treating OA with osteoporosis[1]. Even though alendronate, also a member of the bisphosphonate family, has been shown to prevent subchondral bone lesions and to reduce knee pain in clinical trials, it is unable to stop the progression of OA[19]. Additionally, a recent study Zoledronic acid, in a high-dose regimen, proved to be chondroprotective in a well-established animal model of OA[20]. At present, in the reported preclinical studies, most of the drug treatment programs have been initiated several days before or on the day of OA model establishment[1, 3, 18, 21, 22]. However, the perfect timing of clinical treatment for OA is unpredictable and impractical; that is, it would be impossible to intervene with drug treatments before OA onset. Therefore, drug intervention after the formation of early OA is more clinically meaningful. Studies in this area are rare, and whether PAM is able to prevent or reverse the progression of early-stage OA has not been investigated. Additionally, if there were any effects, would they be associated with changes in the ratio of OPG to RANKL, MMP-9 and TLR-4 in cartilage and/or subchondral bone?

Based on this hypothesis, we established a lapine model of OA induced by anterior cruciate ligament transection (ACLT) to investigate whether PAM could prevent or even reverse subchondral bone loss and cartilage degeneration at the early stage of OA. Moreover, subchondral and cartilaginous OPG, RANKL and relevant metabolic modulators involved in cartilage and subchondral bone remodeling were also investigated.

## Methods

### Ethics statement

This study was carried out in strict accordance with the recommendations in the Guide for the Care and Use of Laboratory Animals of Xu Zhou Medical University. The protocol was approved by the Committee on the Ethics of Animal Experiments of Xu Zhou Medical University (Permit Number: SYXK (su) 2007-0037. All surgery was performed under sodium pentobarbital anesthesia, and all efforts were made to minimize pain and suffering.

### Animals and study design

60 New Zealand white rabbits (8 months old) weighing approximately 2.5 kg were housed singly in cages in sanitary ventilated animal rooms with controlled temperature and humidity and regular light cycles. Before operation, each rabbit was anesthetized by injection of 3% sodium pentobarbital (30 mg/kg) into the ear vein. Each rabbit was positioned supinely on the operation table. After sterile draping, an incision was made in the side of the patella, and additional local anesthesia with lidocaine intramuscular injection for the incision was given, followed by exposure of the articular cavity. Intraoperative protections of cartilage were noted. The right knee joint was shaved and disinfected with isodine. ACLT was performed as described by Yoshioka et al. [23]. A medial parapatellar incision was made. After the patella was dislocated laterally, the knee was flexed maximally so that the anterior cruciate ligament could be readily visualized and identified. It was then transected with a No. 12 blade. An anterior drawing test was performed gently to confirm that the ACL was transected completely. The joint was irrigated with sterile saline and closed. The joint capsule was closed with a running suture of 4–0 nylon thread and the skin incision was closed with running mattress sutures of 3–0 nylon thread. Sham operations were performed in a similar manner, except that anterior cruciate ligament was not transected. After the operation, muscular injection of Bucinnazine (25 mg/d) was administered for postoperative analgesia. Free activity was allowed in the cages without immobilization.

**Figure 1:**
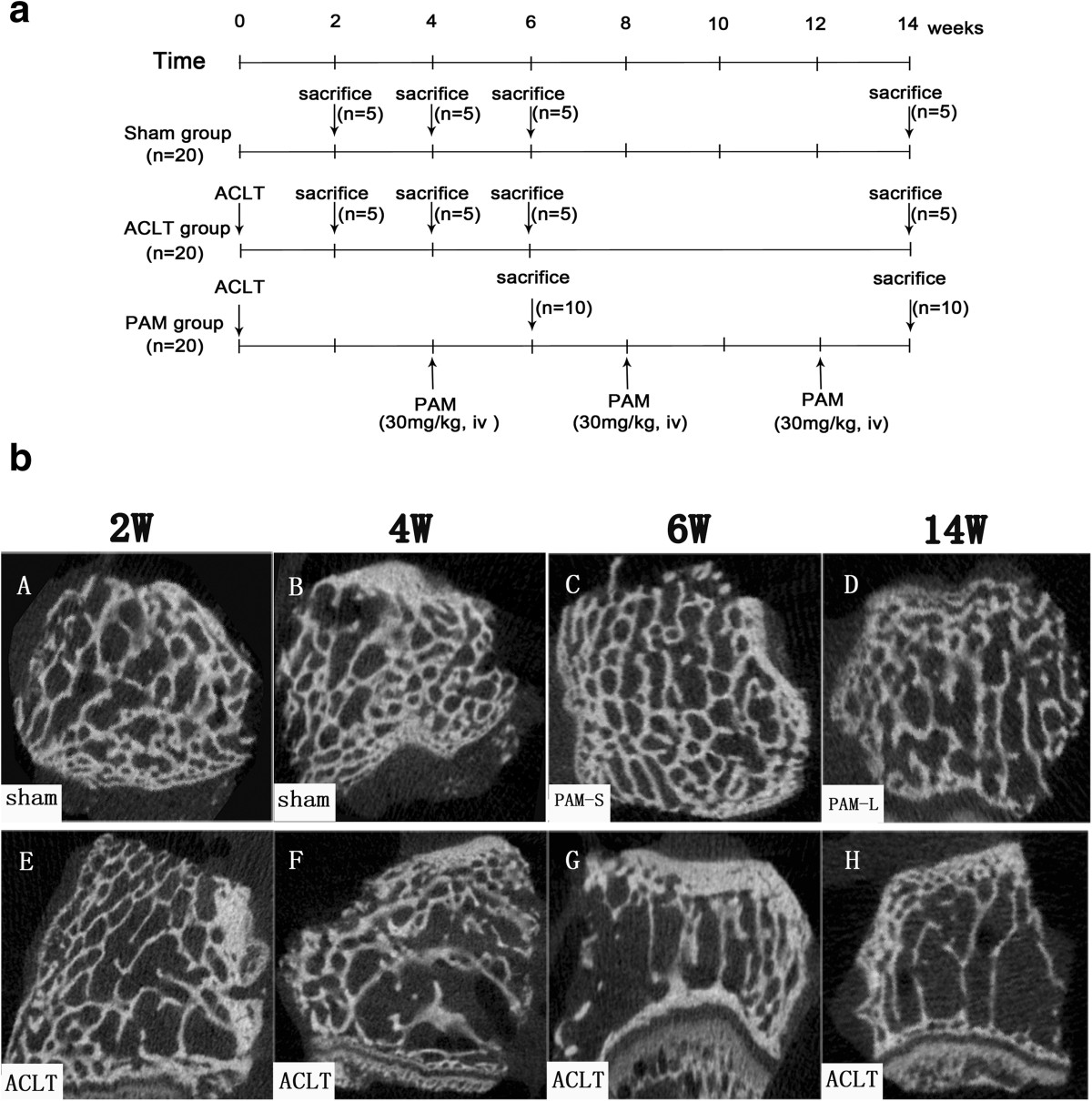
**Experimental design to study the effect of PAM on subchondral bone loss in ACLT- induced osteoarthritis. (a)** Experimental scheme. **(b)** Typical Micro-CT images selected of a highly representative subchondral bone sample for each group. (A,B) Sham-operated group, (C) short-term treatment with PAM, (D) long-term treatment with PAM, (E-H) ACLT-induced osteoarthritis at 2, 4, 6 and 14 weeks. Marked differences in the microarchitecture of subchondral bone were observed.

The rabbits were randomly divided into four groups as follows. Sham-operated with vehicle treatment (Sham group, *n* = 20), OA induced by ACLT with vehicle treatment (ACLT group, *n* = 20), OA-induced ACLT treated with short-term PAM (Sigma, Saint-Quentin Fallavier, France) treatment after ACLT (PAM-S, *n* = 10), and ACLT treated with long-term PAM treatment (PAM-L, *n* = 10). PAM was injected at the 4th week after ACLT in PAM-S and PAM-L groups, and followed by once monthly ear vein injections at a dosage of 3 mg/kg body weight. This dosage was chosen because it can fully improve bone mineral density, osteogenic ability and mechanical properties[24]. In the other groups, only saline infusions of equal volumes were administered. 10 animals were humanely sacrificed at both 2 and 10 weeks after PAM treatment. In the ACLT and Sham groups, five animals were sacrificed at 2, 4, 6, and 14 weeks after model establishment. The experimental scheme is shown in Figure 1a.

### Micro-computerized tomography (micro-CT)

The proximal tibia of each rabbit was scanned and analyzed using the SkyScan1176 Micro-CT system and software (version 1.1; Kontich, Belgium) with the following specifications: voxel size 35 μm, voltage 65 kV, exposure time 250 ms, frame averaging 1, beam filtration filter 1.0 mm aluminum. After scanning, the proximal tibia was three-dimensionally reconstructed using SkyScan software (version 1.1; Kontich). For analysis of the subchondral plate, the load-bearing region (1.04 × 1.04 cm^2^) was selected as the region of interest (ROI). For analysis of subchondral trabecular bone, a cuboid of trabecular bone (1.04 × 1.04 × 1.52 cm^3^) beneath the ROI of the subchondral plate was selected. Bone volume fraction (BV/TV, %), trabecular thickness (TbTh, mm), trabecular number (TbN, 1/mm), trabecular separation (TbSp, mm), trabecular bone pattern factor (TbPf, 1/mm), structure model index (SMI) and degree of anisotropy (DA) were calculated for subchondral trabecular bone.

### Histology and OARSI score

Rabbits were euthanized[25] and the medial condyles of the femurs were fixed with 4% paraformaldehyde (Boster, Wuhan, China) overnight at 4°C on a shaker. Whole medial condyles were decalcified in 14% ethylene-diaminetetraacetic acid for 5 days at 4°C on a shaker. After dehydration by gradient alcohol and infiltration by xylene and paraffin, cartilage samples were embedded in paraffin. Paraffin-embedded joints (4 μm) were sectioned on a sagittal plane. Samples were stained with safranin O (Hengyuan, Shanghai, China) and fast green (Hengyuan) using standard protocols. All sections were evaluated for histologic signs of cartilage degeneration using a modified Osteoarthritis Research Society International (OARSI) scoring system[26].

### Immunohistochemical localization of OPG and RANKL

Paraffin-embedded femur sections (4 μm) were prepared to detect the distribution pattern of cartilaginous and subchondral OPG and RANKL. The sections were incubated with antibodies against OPG and RANKL (Santa Cruz Biotechnology, Dallas, TX, USA). Briefly, tissue sections were washed twice with PBS containing 0.3% Tween 20 for 1 h and incubated with anti-OPG and anti-RANKL polyclonal antibodies, followed by horseradish peroxidase-labeled secondary antibody (Dako, Carpinteria, CA, USA). The color (brown) was developed using 0.5 mg/ml 3,3′-diaminobenzidine tetrahydrochloride. On negative control slides, non-immune goat serum was substituted for the primary antibody. On positive control slides, the brown-yellow precipitate was developed as the OPG and RANKL final product (see Additional file1).

### Western blot analysis

#### Separation of subchondral bone and cartilage

Proximal tibias were frozen in liquid nitrogen then placed in an insulated box filled with liquid nitrogen to avoid protein degradation. Each proximal tibia was then stabilized with a holding clamp. The articular cartilage and epiphyseal plate tissue of each femur condyle sample was cleared away with a microelectronic burnishing instrument (Strong 80, 1 cw power, China) and a drill (SDE-H37L1, Marathon Polishing Hand Grinder, Korea) to isolate the bone and cartilage. During this process, we froze the samples using liquid nitrogen. A dissecting microscope (AXS, Anxi Optical Equipment Manufacturing Co, Shanghai, China) was used to ensure that the articular cartilage and subchondral bone were isolated.

Tissues from cartilage and subchondral bone were homogenized in liquid nitrogen, and protein extracted from the resulting powder using a protein extraction buffer containing 15 mM HEPES, 10% glycerol, 0.5% NP-40, 250 mM NaCl, 1 mM ethylene-diaminetetraacetic acid, 1:1000 PMSF, and a protease inhibitor cocktail (Sigma-Aldrich, St. Louis, MO, USA). Protein determination was carried out as described previously[27], and subsequently, 30 μg of protein from the cartilage and the subchondral bone, respectively, were resolved on 15% polyacrylamide sodium dodecyl sulfate gels. After transfer to polyvinylidene difluoride membranes (Millipore, Molsheim, France) in 48 mM Tris, 39 mM glycine and 20% methanol at 20 V for 1 h at room temperature, membranes were blocked in 5% skimmed milk in PBS-Tween 20 for 1 h at room temperature and incubated overnight at 4°C with anti-RANKL antibodies (sc-7628, 1:1000, Santa Cruz), anti-OPG antibodies (sc-8468, 1:1000, Santa Cruz), anti-MMP9 antibodies (BS-1241, 1:1000, Bioworld Technology, St. Louis Park, MN, USA) and anti-TLR4 antibodies (ab183459, 1:800, Abcam, Cambridge, UK). Antibody binding was detected by enhanced chemiluminescence using peroxidase-labelled secondary antibodies and the results are expressed as arbitrary densitometric units. Loading control was performed on the 15% polyacrylamide-SDS gels using EZBlue Gel Staining Reagent (Sigma-Aldrich).

### Statistical analysis

All data are expressed as the means ± SEM. Statistical analyses were performed using the SPSS statistics package (version 13.0, SPSS Inc., Chicago, IL, USA). The significance of statistical data was analyzed using a two-way analysis of variance (ANOVA) to determine the “time effect” and “time by group interaction effect”. If *F* values were significant, a paired t-test was applied and the *P* values were adjusted for repeated comparisons using Holm’s Bonferroni step-down procedure. A comparison between groups at each time point was performed using a one-way ANOVA with Tukey’s post hoc test. A value of *P* <0.05 was considered statistically significant.

## Results

### Establishment of ACLT-induced osteoarthritis model in rabbits

All operations were performed smoothly without complications. At euthanasia, no significant variation in the body weights between the experimental groups was observed and the complete transection of each anterior cruciate ligament was confirmed grossly. Periarticular areas were generally clear or slightly blood tinged, with no gross signs of inflammation or infection.

### Impact of PAM on the structure of subchondral bone

**Figure 2:**
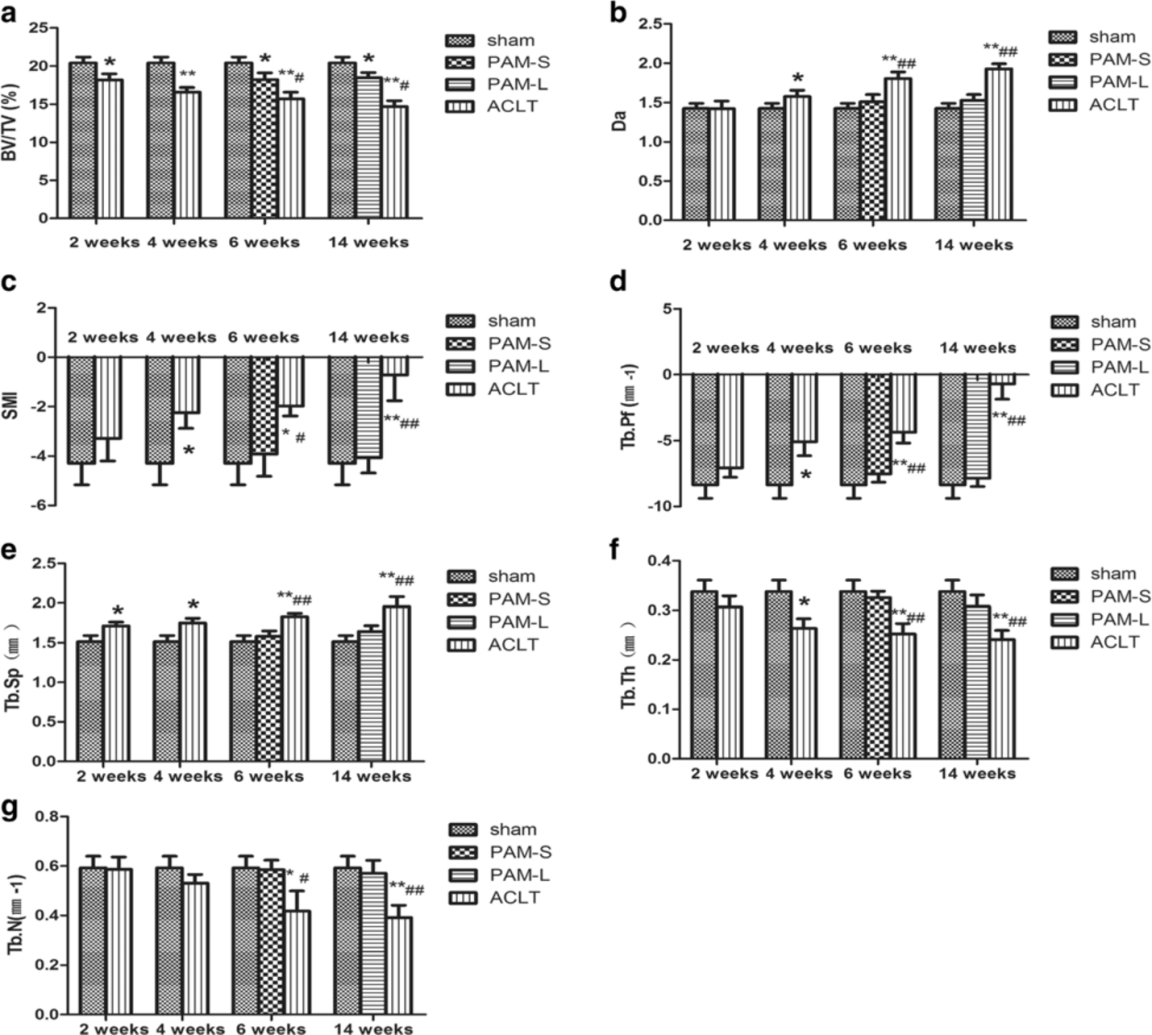
**Quantitative analyses of the subchondral bone morphometric parameters from Micro-CT.** Bone parameters assessed included **(a)** BV/TV, **(b)** DA, **(c)** SMI, **(d)** TbPf, **(e)** TbSp, **(f)** TbTh, **(g)** TbN. * and ** indicate groups significantly different from the sham-operated rabbits treated with vehicle (*P <0.05 and ** *P* <0.01). # and ## indicate significant differences between the ACLT group and rabbits treated with pamidronate. (# *P* <0.05 and ##: *P* <0.01).

Alterations in three-dimensional microstructures of the subchondral bone are presented in Figure 1b. At 2 weeks after model establishment, the ACLT group showed lower BV/TV percentages, but higher TbSp, compared with the Sham group (*P* <0.05, Figure 2). At 4 weeks after model establishment, BV/TV and TbTh were both significantly lower (*P* <0.05, Figure 2), but TbPf, SMI, TbSp and DA were all significantly increased compared with the Sham group (*P* <0.05, Figure 2). TbN consistently decreased over the first 6 weeks after model establishment (*P* <0.05, Figure 2), and these parameters changed continuously in the same direction throughout the study period. After 2 weeks of treatment, the PAM group showed significantly increased BV/TV, TbN and TbTh compared with the ACLT group (*P* <0.05 or *P* <0.01); however, TbPf, TbSp, DA and SMI were all significantly lower (*P* <0.05 or *P* <0.01, Figure 2). After 10 weeks of PAM treatment, with the exception of the slightly lower BV/TV percentage (*P* <0.05), other parameters changed insignificantly compared with the Sham group (Figure 2). The data indicate that at 2 weeks after the establishment of ACLT-induced OA in rabbits, changes in microstructures of the subchondral bone had started to appear. At 4 weeks after model establishment, related parameters in the subchondral bone were altered significantly via treatment with PAM, indicating that structures of the subchondral bone at the early stage of OA had significantly improved.

### Impact of PAM on the OARSI scores in the lapine ACLT-induced model OA

**Figure 3:**
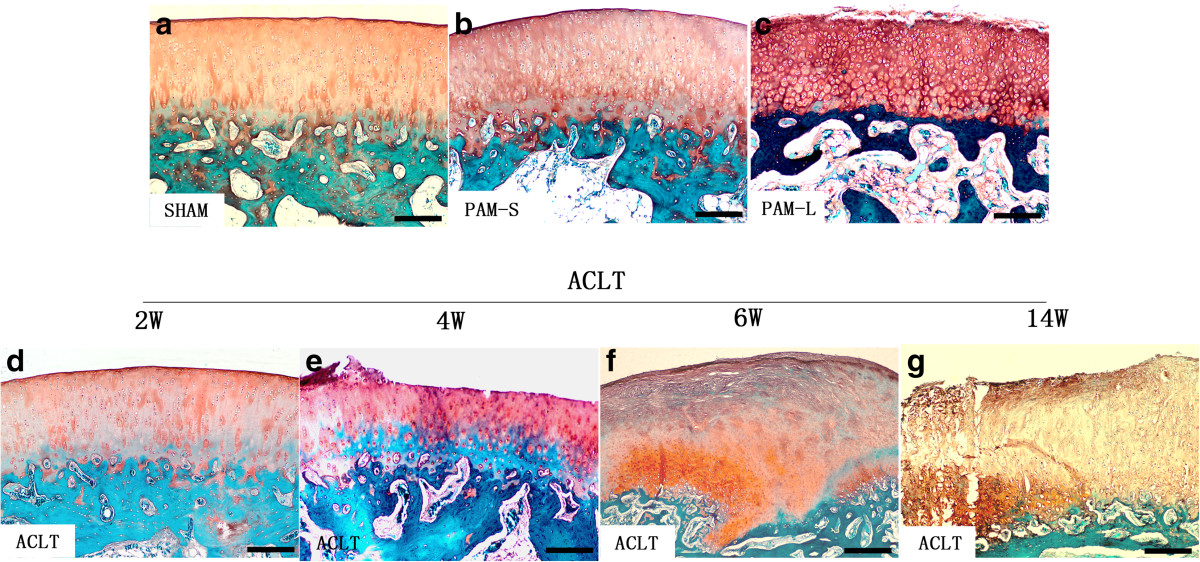
**Effects of PAM treatment on cartilage surface erosion.** The sections were stained with safranin-O (red stain) for glycosaminoglycans, rapid green for bone. **(a)** The Sham group had no erosion-like changes in the cartilage surface. **(b,c)** Short-term and long-term treatment with PAM significantly increased chondrocytes and matrices, while fibroses of the cartilage surface were significantly reduced compared with the ACLT group at 6 and 14 weeks. **(d)** ACLT group at 2 weeks, no significant changes were observed in the cartilage surface. **(e)** ACLT group at 4 weeks, staining of safranin O had been partially lost and various degrees of surface fibrosis, cracks, cell loss proliferation were observed in the surface layer of the hyaline cartilage. **(f,g)** ACLT group at 6 and 14 weeks, chondrocytes of the hyaline cartilage surface were massively reduced, fibroses were extensive, osteophytes had formed, vertical fractures had increased and safranin O staining in the cartilaginous area was significantly reduced. Bar represents 200 μm.

**Figure 4:**
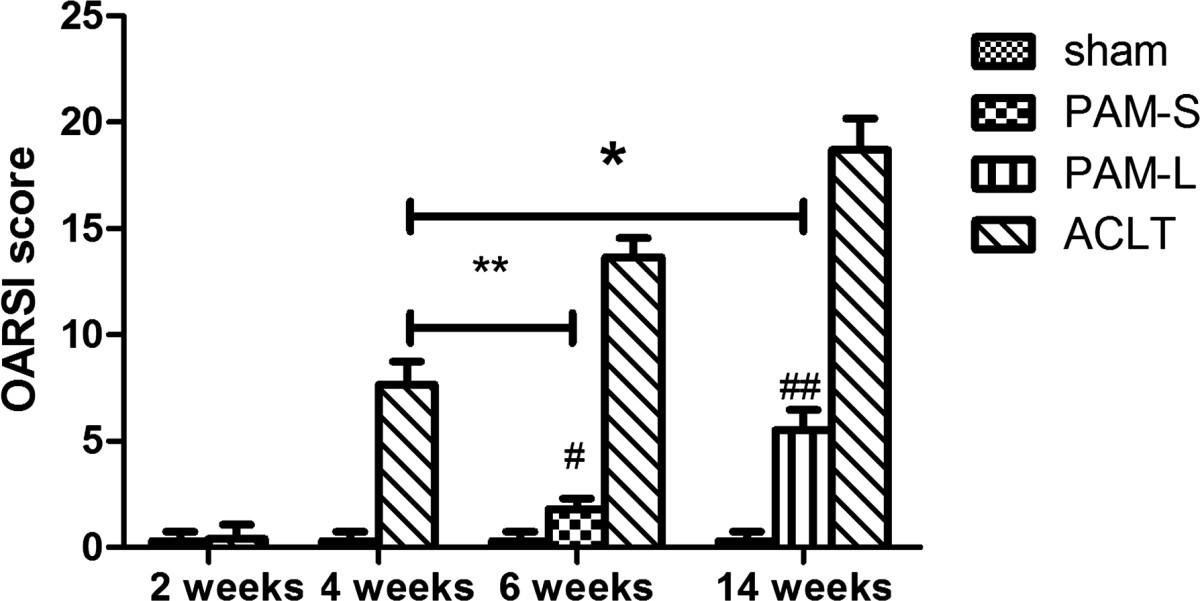
**Assessment of articular cartilage changes according to the 2010 OARSI recommendations in the rabbit.** The score represents the sum of the scores of staining, structure, chondrocyte density and cluster formation. A significant increase of OARSI scores in the ACLT group can be observed with time, and PAM can prevent and lower the increase in the OA scores, compared with the ACLT group at 4 weeks. *P <0.05, **P <0.01, # indicates group significantly different from the sham-operated rabbits treated with vehicle (# *P* <0.05 and ## *P* <0.01).

Safranin O and fast green staining of cartilage tissue detected no erosion-like changes in the cartilage surface at any time in the Sham group (Figure 3a). In the ACLT group, no significant changes were observed either in the cartilage surface at 2 weeks after model establishment (Figure 3d). However, at 4 weeks after model establishment, staining of safranin O had been partially lost, indicating proteoglycan loss in the articular cartilage (Figure 3e). Various degrees of surface fibrosis, cracks, cell loss and proliferation were observed in the surface layer of the hyaline cartilage. In the ACLT group at 6 and 14 weeks after model establishment, the following observations were recorded: chondrocytes of the hyaline cartilage surface were markedly reduced, fibrosis was extensive, osteophytes had formed, vertical fractures had increased and safranin O staining in the cartilaginous area was significantly reduced (Figure 3f and g). Compared with the ACLT group, at 2 and 10 weeks after PAM treatment, chondrocytes and matrices in the PAM group were significantly increased, fibroses of the cartilage surface were significantly reduced, and no increase in fractures was observed (Figure 3b and c). Moreover, OARSI scores indicated that PAM prevented and attenuated an increase in OA (*P* <0.05 or *P* <0.01, Figure 4). These results indicate that PAM treatment in the PAM-S and PAM-L groups both prevented OA alterations in chondrocyte morphology and losses in the cartilaginous matrix, or that PAM treatment may have reversed the pathological lesions of the articular cartilage (Figure 3).

### Correlations between cartilage and bone changes

**Figure 5:**
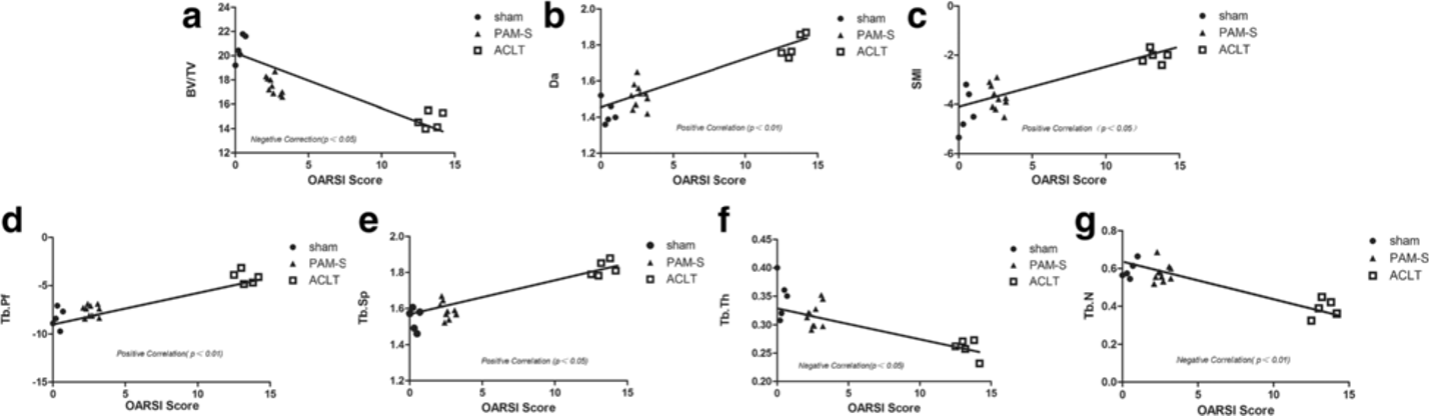
**Correlations between the OARSI score and the micro-CT quantitative bone parameters at 6 weeks. (a)** BV/TV, **(b)** DA, **(c)** SMI, **(d)** TbPf, **(e)** TbSp, **(f)** TbTh, **(g)** TbN. Significant negative correlations were identified between OARSI scores and BV/TV, TbTh and TbN, and significant positive correlations were identified between OARSI scores and TbSp, TbPf, SMI and DA at 6 weeks. *P* values are indicated on a where statistically significant. The symbols represent the different specimens (● = Sham group, ▲ = PAM-S group, □ = ACLT group)

Associations between OARSI scores and the micro-CT bone structure assessments at 6 weeks and 14 weeks are illustrated in Figures 5 and6. Significantly negative correlations were identified between OARSI scores and BV/TV percentage (*P* <0.05), TbTh (*P* <0.05), and TbN (*P* <0.01), but significantly positive correlations were identified for TbSp (*P* <0.05), TbPf (*P* <0.01), SMI (*P* <0.05), and DA (*P* <0.01). Importantly, the subchondral bone changes correlated with the cartilage lesions, providing additional evidence that subchondral bone is involved and plays a critical role in the pathogenesis of ACLT-induced OA.

**Figure 6:**
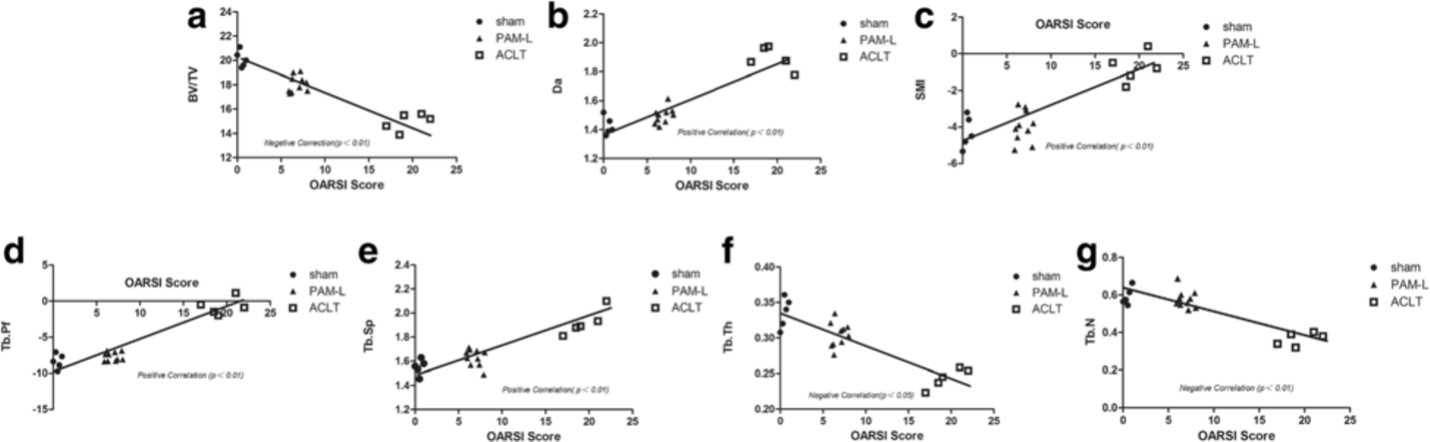
**Correlations between the OARSI score and the Micro-CT quantitative bone parameters at 14 weeks. (a)** BV/TV, **(b)** DA, **(c)** SMI, **(d)** TbPf, **(e)** TbSp, **(f)** TbTh, **(g)** TbN. Significant negative correlations were identified between OARSI scores and BV/TV, TbTh and TbN, and significant positive correlations were identified between OARSI scores and TbSp, TbPf, SMI and DA at 14 weeks. *P* values are indicated on **b** where statistically significant. The symbols represent the different specimens (● = Sham group, ▲ = PAM-L group, □ = ACLT group).

### Impact of PAM on OPG and RANKL expression in subchondral bone and cartilage

**Figure 7:**
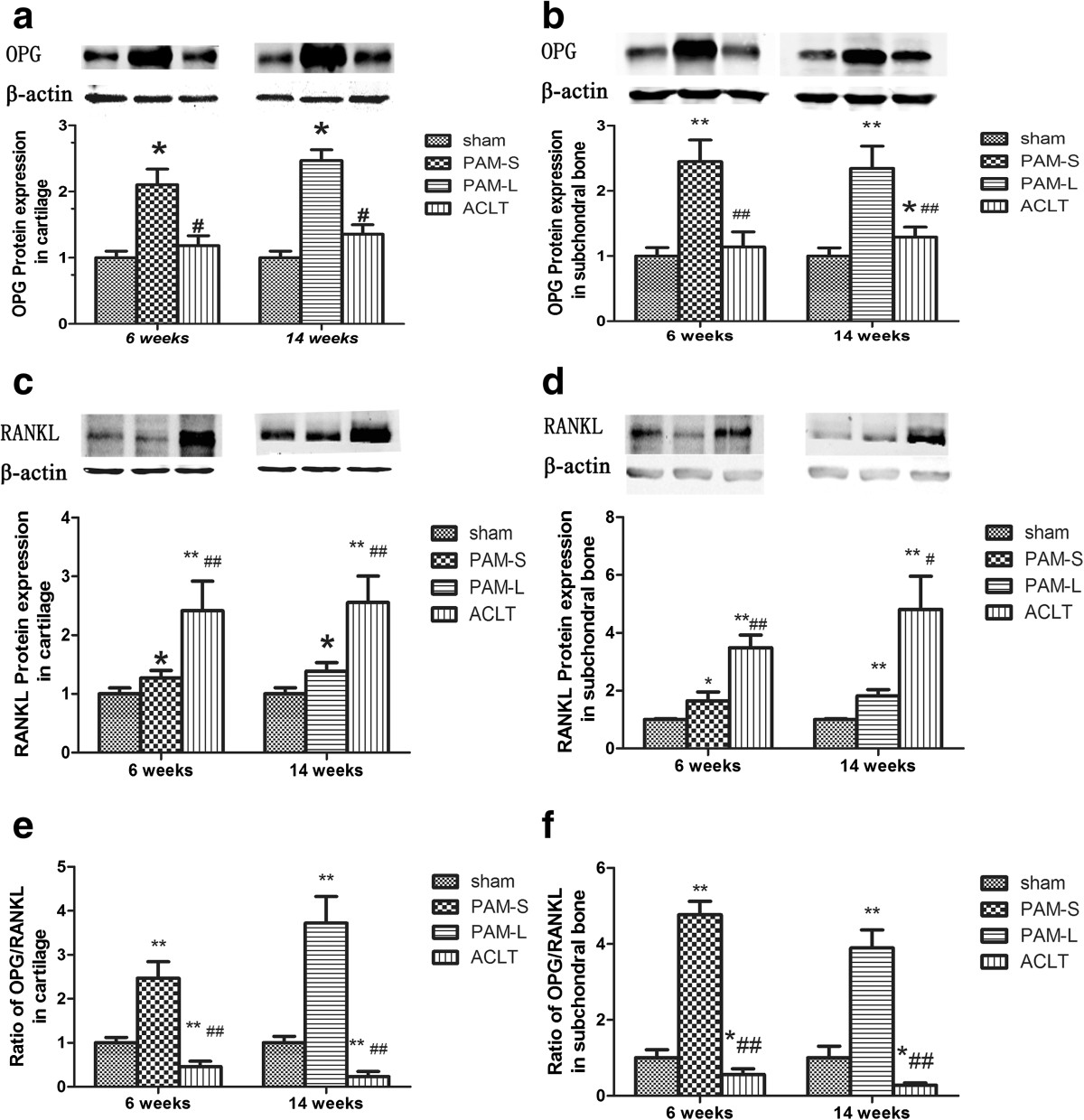
**Effects of PAM treatment on OPG and RANKL protein expression and OPG/RANKL ratio in cartilage and subchondral bone.** Relative levels measured by western-blotting are shown, **(a)** OPG protein expression in cartilage, **(b)** OPG protein expression in subchondral bone, **(c)** RANKL protein expression in cartilage, **(d)** RANKL protein expression in subchondral bone, **(e)** Ratio of OPG/RANKL in cartilage, **(f)** Ratio of OPG/RANKL in subchondral bone. * and ** indicate groups significantly different from the sham-operated rabbits treated with vehicle (*P <0.05 and **P <0.01). # and ## indicate significant differences between the ACLT group and rabbits treated with pamidronate. (# *P* <0.05 and ## *P* <0.01).

**Figure 8:**
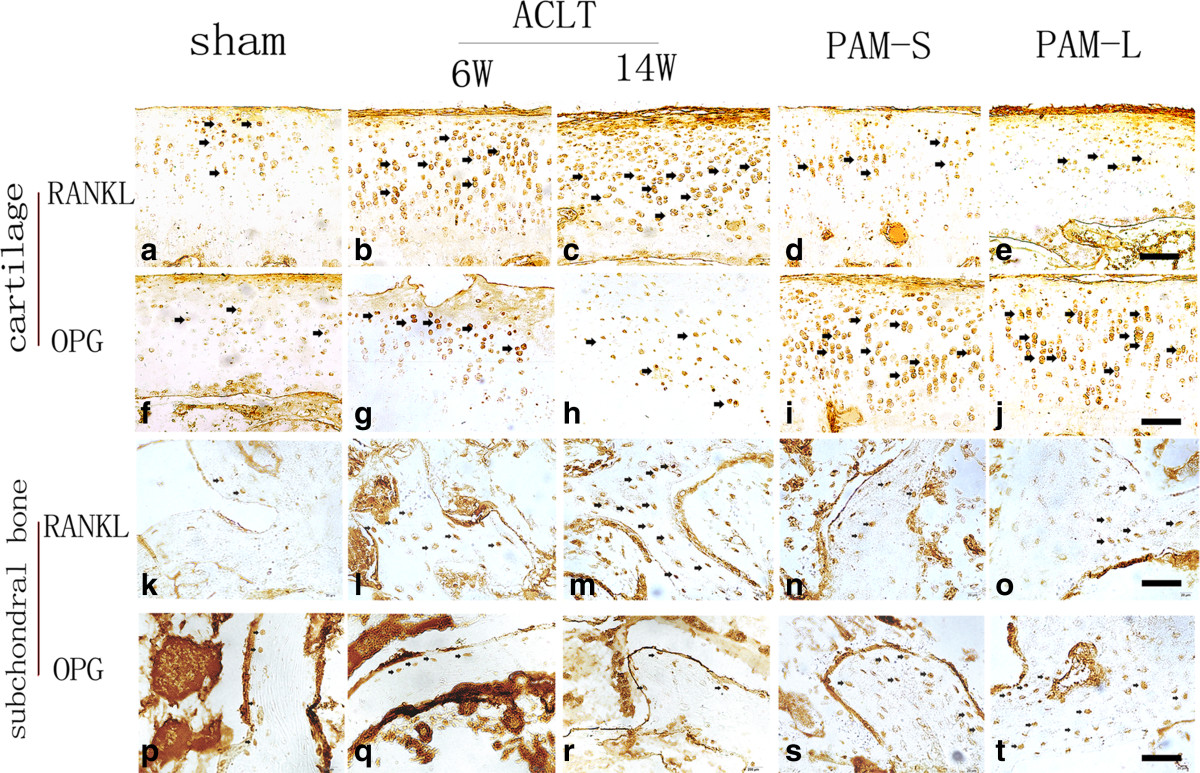
**Effects of PAM treatment on expression of RANKL and OPG in the cartilage and subchondral bone. (a–e)** Immunohistochemical detection of RANKL expression in cartilage in Sham **(a)**, ACLT **(b,c)** and PAM **(d,e)** samples, arrows point to positive cells. **(f–j)** OPG positive cells in the cartilage of the indicated samples. **(k–o)** RANKL positive cells in the subchondral bone samples. **(p–t)** OPG positive cells in the subchondral bone samples. Bar represents 100 μm.

Western blot indicated that protein expression of OPG and RANKL was present in cartilage and subchondral bone tissue in all groups (Figure 7). Compared with the Sham group, protein expression of OPG and RANKL in cartilage and subchondral bone tissues in the ACLT group were both significantly higher (*P* <0.05 and *P* <0.01), although the increase in RANKL expression was more significant than that of OPG (Figure 7). Therefore, the ratios of OPG/RANKL in cartilage and subchondral bone in the ACLT group were lower than in the Sham group (*P* <0.01, Figure 7e and f). However, protein expression of OPG in the articular cartilage and subchondral bone in both PAM groups were significantly higher when compared with either the Sham group or the ACLT group (*P* <0.01, Figure 7a and b). Compared with the ACLT group, expression of RANKL in both PAM groups was lower (Figure 7c and d). Hence, the OPG/RANKL ratios in the PAM group were significantly higher, compared either with those of the Sham or ACLT groups (*P* <0.01, Figure 7e and f).Immunohistochemical analyses detected low expression of RANKL and OPG in cartilaginous chondrocytes and subchondral bone osteoblasts in the Sham group (Figure 8a, f, k and p). In the ACLT group, OPG-positive cells were slightly more numerous (Figure 8g, h, q and r), and RANKL-positive cells were significantly more numerous (Figure 8b, c, l and m), respectively. Compared with the ACLT group, OPG-positive cells were significantly more numerous in both articular cartilage and subchondral bone in both PAM groups (Figure 8i, j, s and t). Furthermore, the numbers of RANKL-positive cells in both articular cartilage and subchondral bone were also significantly lower (Figure 8d, e, n and o). These results indicate that PAM significantly increased the OPG/RANKL ratio in this lapine model of ACLT-induced OA. Therefore, these immunohistochemical results also confirm the western blot and micro-CT results.

### Impact of PAM on the expression of MMP-9 and TLR4 in subchondral bone and cartilage

**Figure 9:**
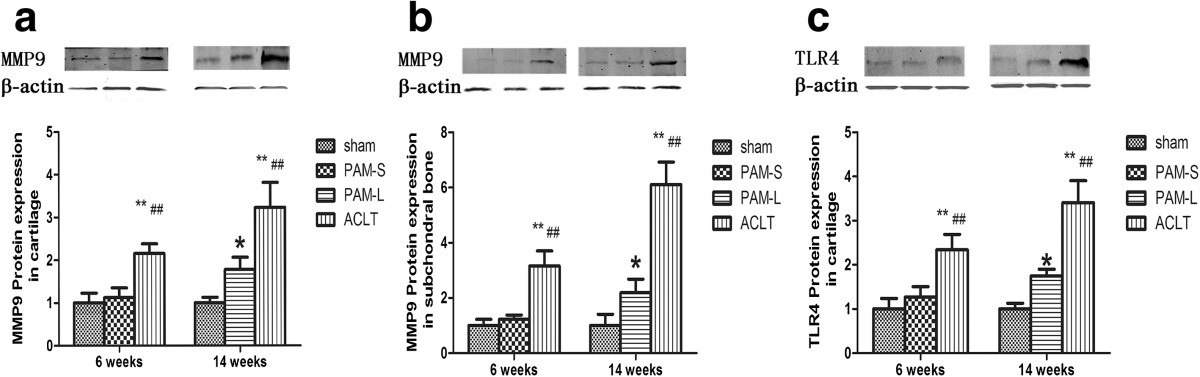
**Effect of PAM treatment on MMP9 and TLR4 protein expression in cartilage and subchondral bone.** Relative protein levels as measured by western blot are shown, **(a)** MMP9 protein expression in cartilage and **(b)** subchondral bone. **(c)** TLR4 protein expression in cartilage. * and ** indicate groups significantly different from the sham-operated rabbits treated with vehicle (*P <0.05 and **P <0.01). # and ## indicate significant differences between the ACLT group and rabbits treated with pamidronate. (# *P* <0.05 and ## *P* <0.01).

Western blot results showed low expression of MMP-9 and TLR4 in the Sham group (Figure 9). In contrast to the Sham group, expression of MMP-9 and TLR4 was significantly higher in the ACLT group (*P* <0.01, Figure 9). Notably, compared with the ACLT group, expression of MMP-9 and TLR4 in the PAM treated groups was significantly down-regulated (*P* <0.01). These data indicate that timely treatment with PAM can counteract subchondral bone degradation as determined by MMP-9 protein expression and can reduce the cartilage destruction and inflammation associated with TLR4 activation. Therefore, these western blot results are also in accord with the histology and micro-CT results.

## Discussion

OA is mainly characterized by wearing of the articular cartilage and abnormal remodeling in the subchondral bone[1]. To date, its pathogenesis remains unknown and there is no known cure. Some studies indicate that increased subchondral bone remodeling could lead to cartilage degeneration initially[1, 28]. Mechanically, the subchondral bone supports the overlying cartilage and absorbs the forces transmitted by the joint. Mineralization of the subchondral bone and increased rigidity reduce its buffering and load balancing capacities and thus accelerate the cartilage degeneration process[3, 29]. However, some scholars have posited that changes in subchondral bone could exist during or after cartilaginous degeneration[10]. Therefore, whether the development of OA originates from cartilage or subchondral bone alterations remains controversial.

In the present study, at 2 weeks after model establishment, the BV/TV of the subchondral bone was significantly reduced and the TbSp was increased, indicating that bone loss had begun in this ACLT-induced OA model. However, at 2 weeks after model establishment, the OARSI score of the articular cartilage in the ACLT group was not significantly different from that of the Sham group (*P >* 0.05, Figure 4). Therefore, subchondral bone changes preceded the changes in the articular cartilage in this OA model.

To explore the methods of treatment to protect against articular cartilage deterioration and subchondral bone loss, bone anticatabolic agents such as bisphosphonates may be used as a means to inhibit the subchondral bone resorption[1–4]. Although limited clinical researches have shown beneficial effects of bisphosphonates on subchondral bone and prevent the progression of OA, there is no study evaluating a selected sample of patients with early OA owing to ethical reasons. In a 2-year trial, risedronate, also a member of bisphosphonate family, decreased biochemical markers of cartilage degeneration and partial symptoms, but did not prevent the radiographic progression of advanced OA[30]. Furthermore, the majority of bisphosphonates treatment animal programs have been initiated several days before or on the day of OA animal model establishment[1, 3, 20–22]. The aforementioned studies suggest that further studies are necessary to evaluate the importance of timing of treatment with anti-resorptive agents in the early stage of OA.

In this study, treatment with PAM was initiated at 4 weeks after OA establishment. Compared with the ACLT group, BV/TV, TbN and TbTh were all significantly higher, while TbPf, TbSp, Da and SMI were all significantly lower in the PAM treated groups (Figure 2). After 2 weeks of PAM treatment, loss of subchondral bone was significantly reduced and osteogenic ability was significantly improved. After 10 weeks of PAM treatment, only BV/TV was reduced (*P* <0.05) compared with the Sham group (Figure 2). These results indicate that PAM treatment stopped bone loss in the subchondral bone and even reversed the pathological lesions in the subchondral bone after long-term therapy.

We used the ACL transection rabbit model for the study of cartilage lesions, as it reproduces all of the OA-associated lesions in arthrosis. Moreover, the ACLT model is a significant risk factor for development of post-traumatic osteoarthritis, which loses the proteoglycan and collagen molecules from cartilage in the first few weeks after anterior cruciate ligament injury[31]. The response of cartilage lesions to therapy can be graded according to an adapted histological and histochemical grading system (OARSI score) that is evaluated via staining for structure, chondrocyte density and cluster formation[26, 32]. In this study, at 4 weeks after model establishment, safranin O staining in the ACLT group was partially lost and fibroses were observed on the surface of hyaline cartilage (Figure 3), indicating loss of articular cartilage proteoglycan and induction of early OA. Thereafter, OA-like signs of massive reduction of chondrocytes, loss of matrices, extensive fibroses, and formations of osteophyte and vertical cracks appeared at 6 and 14 weeks (Figure 3f and g)[33]. However, both PAM groups stopped or improved the morphological changes in chondrocytes and loss of cartilage matrix in early-stage OA and OARSI scores in the PAM group were significantly lower than those of the ACLT group (*P* <0.01, Figure 4). More importantly, both short- and long-term treatments attenuated the articular lesions generated (Figures 3,4). Moreover, excellent correlations were established between parameters of microstructure in subchondral bone and the cartilage damage score. Significant negative correlations were identified between OARSI scores and BV/TV, TbTh and TbN, and significant positive correlations were identified between OARSI scores and TbSp, TbPf, SMI and DA (Figures 5 and6). Thus, these data provide further support that subchondral bone loss and cartilage lesions are closely related. Specifically, the improvement of microstructure and subchondral bone remodeling following treatment with PAM would markedly contribute to the avoidance of progression of cartilage damage in our experimental model.

To elucidate the mechanisms by which PAM protects against articular cartilage and subchondral bone erosion in OA, we investigated the RANKL/OPG system in both subchondral bone and cartilage. It is reported that in a rat knee model of osteoporosis, alendronate treatment at the early or late stages could both raise the ratio of OPG to RANKL[3]. However, Reyes-Garcia et al. measured serum levels of OPG and RANKL in women receiving alendronate treatment for postmenopausal osteoporosis and found that total serum RANKL levels increased during the one-year treatment[34]. Moreover, a recent study has demonstrated that OPG could reduce aggrecan cleavage and cartilage proteoglycan release and then represent the protective role in the regulation of cartilage catabolism[1]. In this study, western blot and immunohistochemistry analysis indicated that the ratio of OPG to RANKL in both cartilage and subchondral bone was significantly increased after short- and long-term treatments with PAM in ACLT-induced OA (Figures 7 and8). Obviously, the results suggest that PAM treatment could upregulate OPG expression, while downregulating RANKL expression in both subchondral bone and cartilage.

Since the central clinicopathological features of the progression of OA are inflammation and articular cartilage destruction, MMP-9 was considered an important proteinase to investigate as it can degrade all components of the complex extracellular matrix in terms of cartilage degradation[16]. Furthermore, many studies have shown that upregulation of MMP-9 results in destruction of articular cartilage in OA patients[35, 36]. TLRs are an important type of pathogen receptor of the innate immune system that have drawn much attention in the study of OA in recent years[37, 38]. Some studies have shown that TLR4 is involved in AGE-induced chondrocyte inflammation in OA in humans[15]. Moreover, one study showed that TLR-4 was capable of regulating the early onset of joint inflammation and cartilage destruction in a murine model of immune complex-mediated arthritis and was closely associated with cartilage destruction[37]. Thus, we detected the expression of MMP-9 and TLR-4 by western blot. Interestingly, we found that the expression of MMP-9 and TLR4 in the ACLT group was significantly higher compared with the Sham group. However, expression of MMP-9 and TLR4 in both PAM treated groups was downregulated compared with the ACLT group (*P* <0.01, Figure 9). Thus, we considered the results to at least provide an additional explanation regarding the control of joint inflammation and mild degeneration in the knee joint of the rabbit after PAM treatment.

## Conclusions

We successfully established an ACLT-induced OA animal model in rabbits. First, we verified the important roles of subchondral bone loss in the pathogenesis of OA and its early incidence before cartilaginous degeneration. Meanwhile, we also found that intervention with PAM at 4 weeks after ACLT-induced OA establishment inhibited subchondral bone loss. Continuous treatment may have reversed the pathological changes in subchondral bone, thus alleviating the process of cartilaginous degeneration. This protection occurred via the upregulation of OPG expression and inhibition of RANKL and MMP-9 expression in the cartilage and subchondral bone, and TLR-4 expression in the cartilage.

## Electronic supplementary material

Additional file 1:**PDF file containing the expressions of OPG and RANKL in positive control slides and negative control slides via immunohistochemical detection.** **(A,D)** Immunohistochemical detection of OPG expression in positive control slides in cartilage **(A)**, and subchondral bone **(D)**. **(B)** RANKL positive cells in the cartilage of the positive control slides. **(E)** RANKL positive cells in the subchondral bone of the positive control slides. **(C)** Negative control slides in cartilage. **(F)** Negative control slides in subchondral bone. (PDF 3 MB)

Below are the links to the authors’ original submitted files for images.

Authors’ original file for figure 1

Authors’ original file for figure 2

Authors’ original file for figure 3

Authors’ original file for figure 4

Authors’ original file for figure 5

Authors’ original file for figure 6

Authors’ original file for figure 7

Authors’ original file for figure 8

Authors’ original file for figure 9

## Abbreviations

ACLT: Anterior cruciate ligament transaction
BV/TV: Bone volume fraction
DA: Degree of anisotropy
MMP-9: Metalloproteinase-9
OA: Osteoarthritis
OPG: Osteoprotegerin
PAM: Pamidronate disodium
PAM-S: ACLT with short-term PAM treatment
PAM-L: ACLT with long-term PAM treatment
RANKL: Receptor activator of nuclear factor κB ligand
SMI: Structure model index
TLR-4: Toll-like receptor-4
TbN: Trabecular number
TbSp: Trabecular separation
TbPf: Trabecular bone pattern factor
TbTh: Trabecular thickness.
