# Supplementary figures and images for: Effects of pamidronate disodium on the loss of osteoarthritic subchondral bone and the expression of cartilaginous and subchondral osteoprotegerin and RANKL in rabbits
Source: BMC Musculoskelet Disord. 2014 Nov 6;15:370. doi: 10.1186/1471-2474-15-370 (PMC4240862; doi:10.1186/1471-2474-15-370)

positive control  
OPG → RANKL

negative control

cartilage

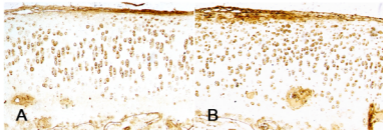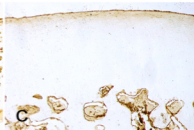

subchondral  
bone

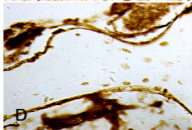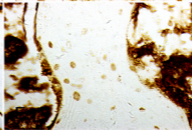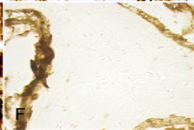

Supplement: Supplementary file 1 — Additional file 1:PDF file containing the expressions of OPG and RANKL in positive control slides and negative control slides via immunohistochemical detection. (A,D) Immunohistochemical detection of OPG expression in positive control slides in cartilage (A), and subchondral bone (D). (B) RANKL positive cells in the cartilage of the positive control slides. (E) RANKL positive cells in the subchondral bone of the positive control slides. (C) Negative control slides in cartilage. (F) Negative control slides in subchondral bone. (PDF 3 MB) [file 12891_2014_2308_MOESM1_ESM.pdf]

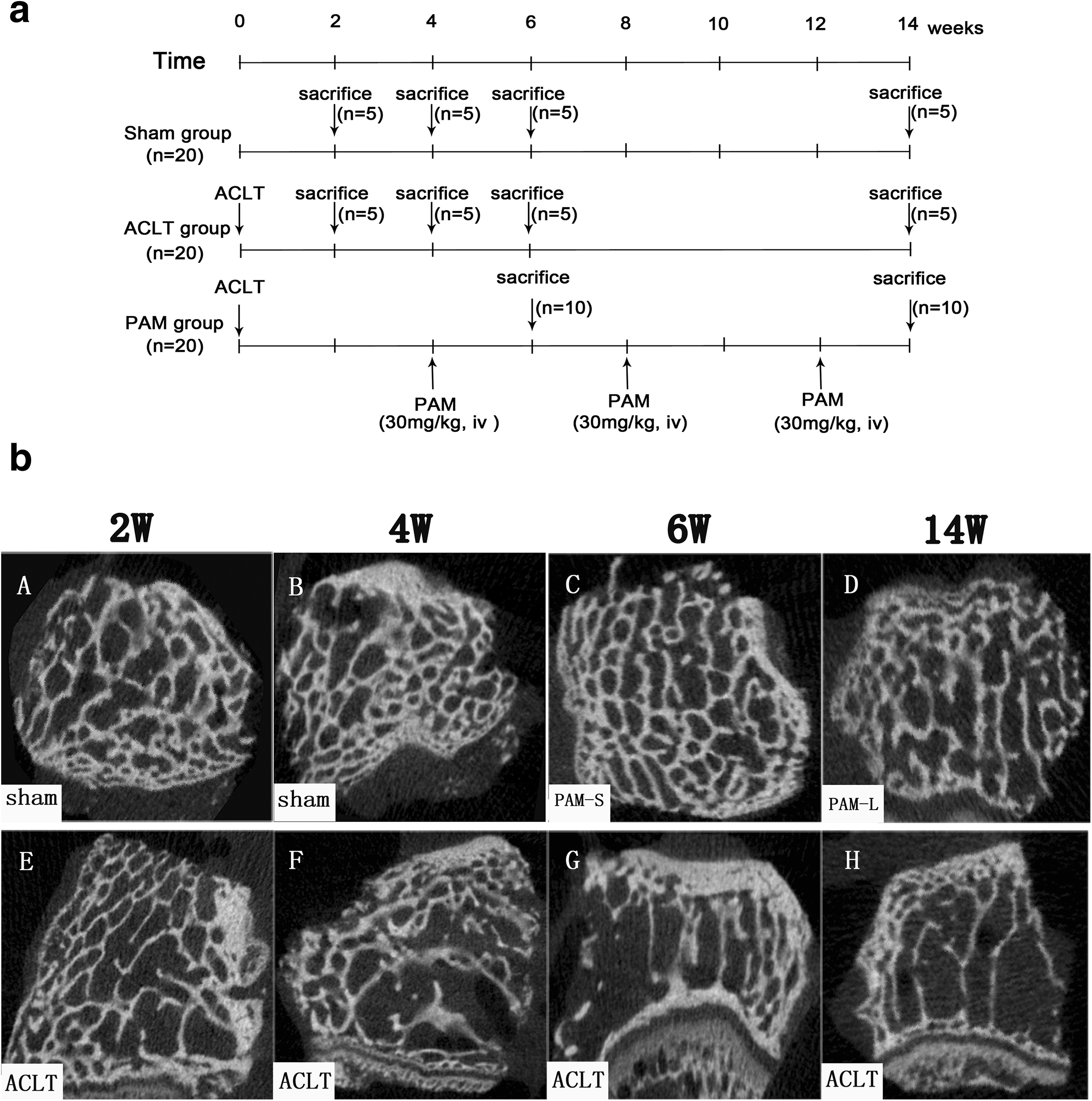

Supplement: Supplementary file 2 — Authors’ original file for figure 1 [file 12891_2014_2308_MOESM2_ESM.tiff]

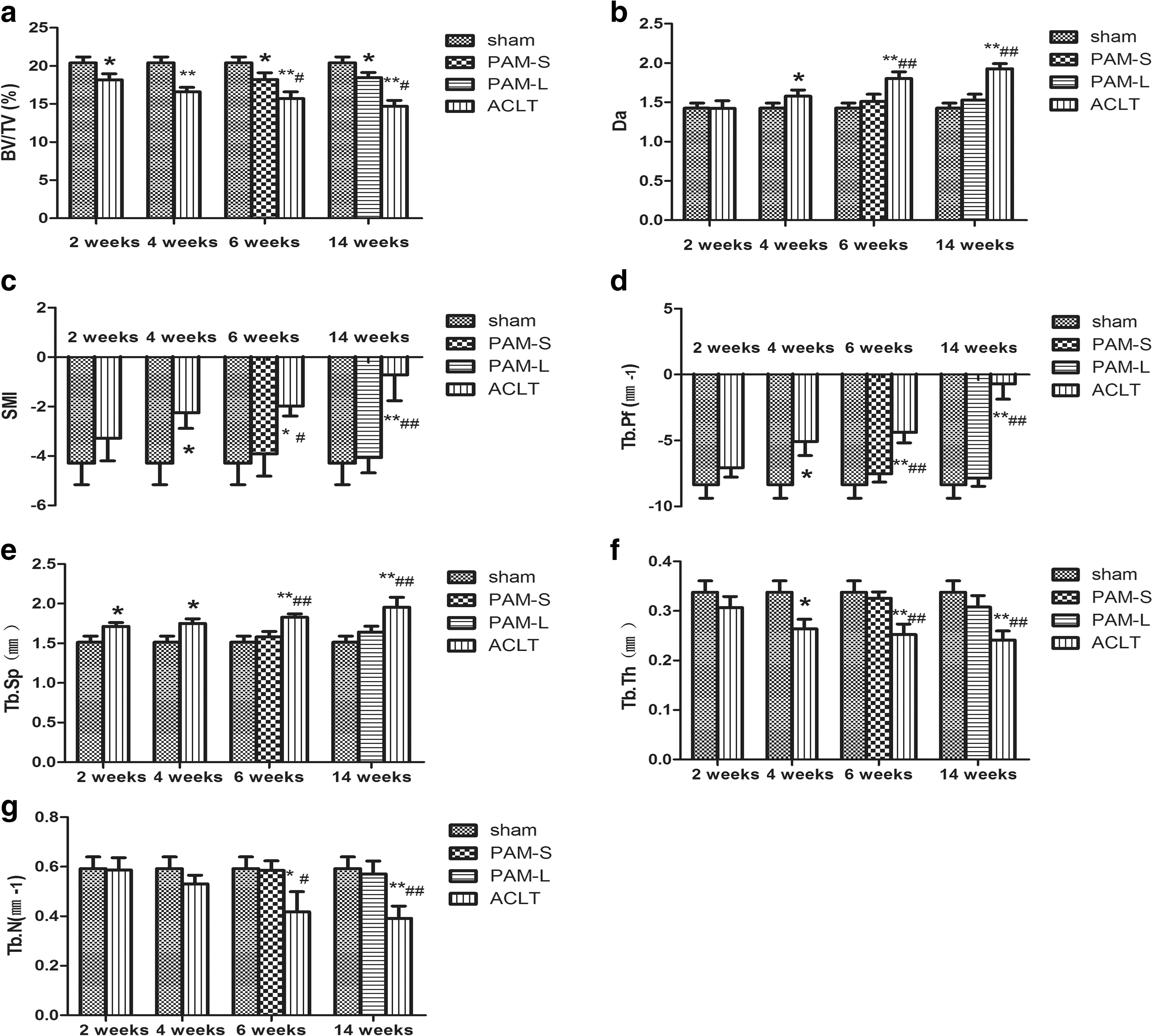

Supplement: Supplementary file 3 — Authors’ original file for figure 2 [file 12891_2014_2308_MOESM3_ESM.tiff]

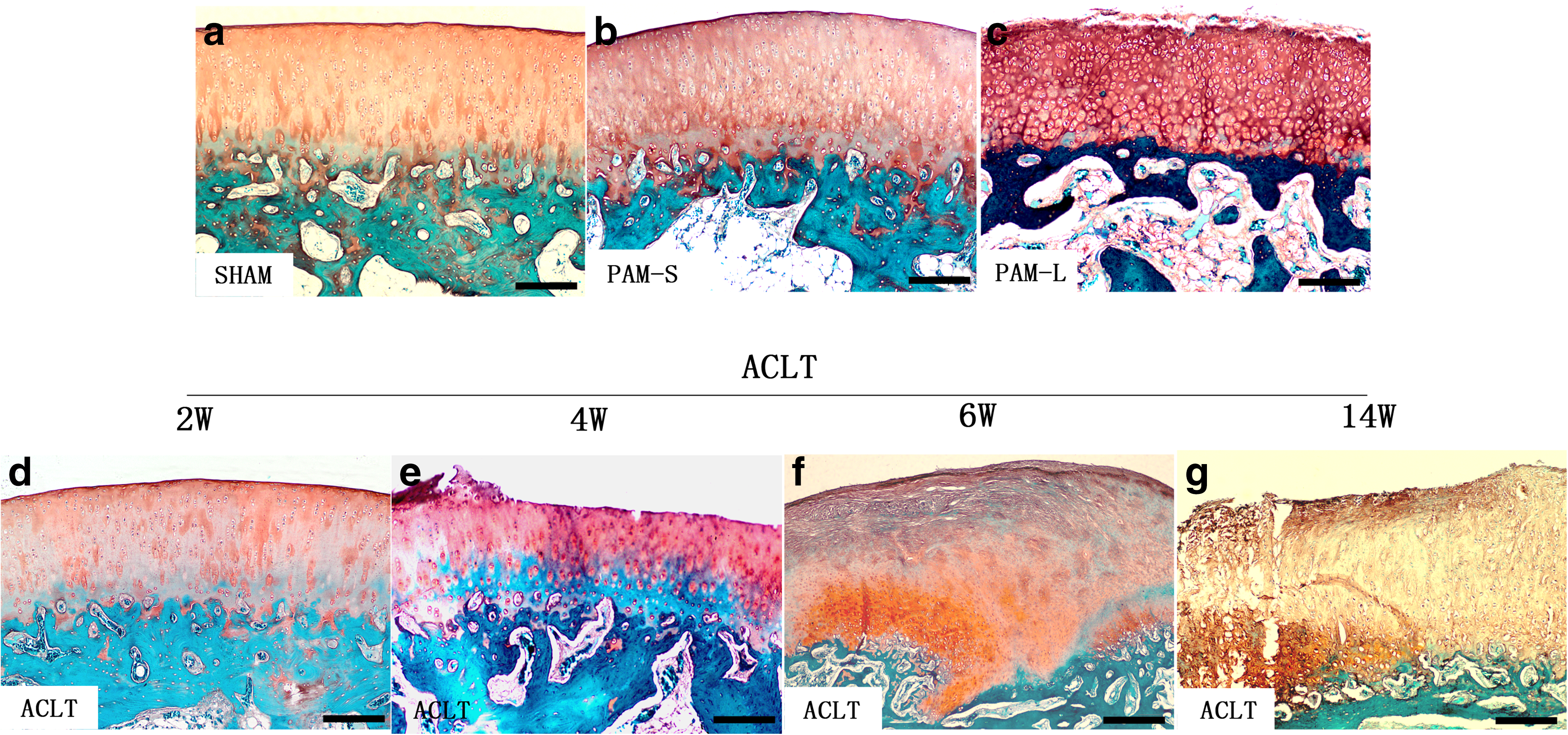

Supplement: Supplementary file 4 — Authors’ original file for figure 3 [file 12891_2014_2308_MOESM4_ESM.tiff]

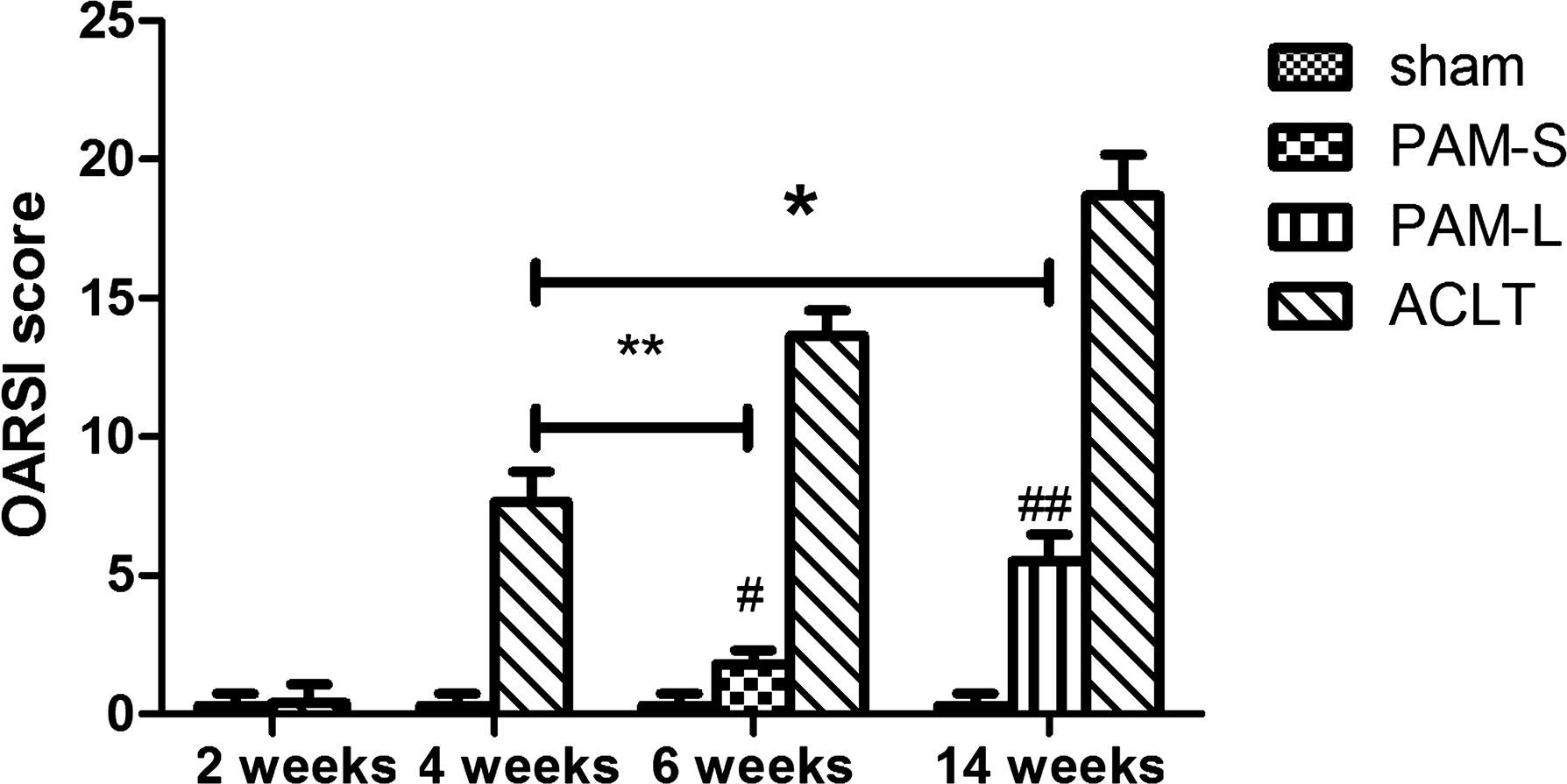

Supplement: Supplementary file 5 — Authors’ original file for figure 4 [file 12891_2014_2308_MOESM5_ESM.tiff]

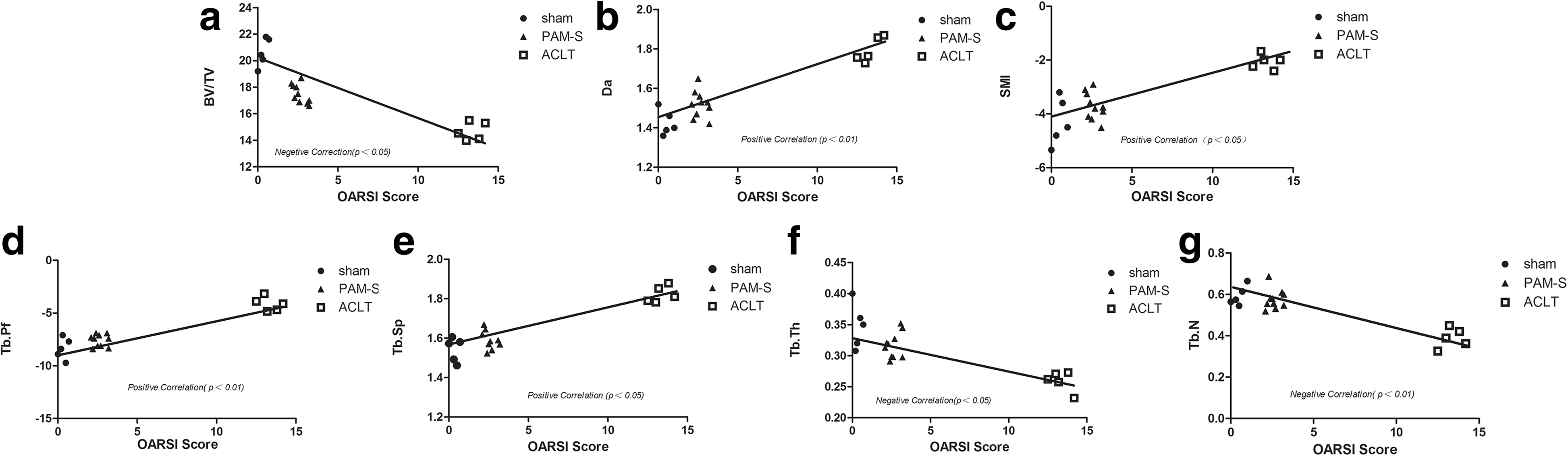

Supplement: Supplementary file 6 — Authors’ original file for figure 5 [file 12891_2014_2308_MOESM6_ESM.tiff]

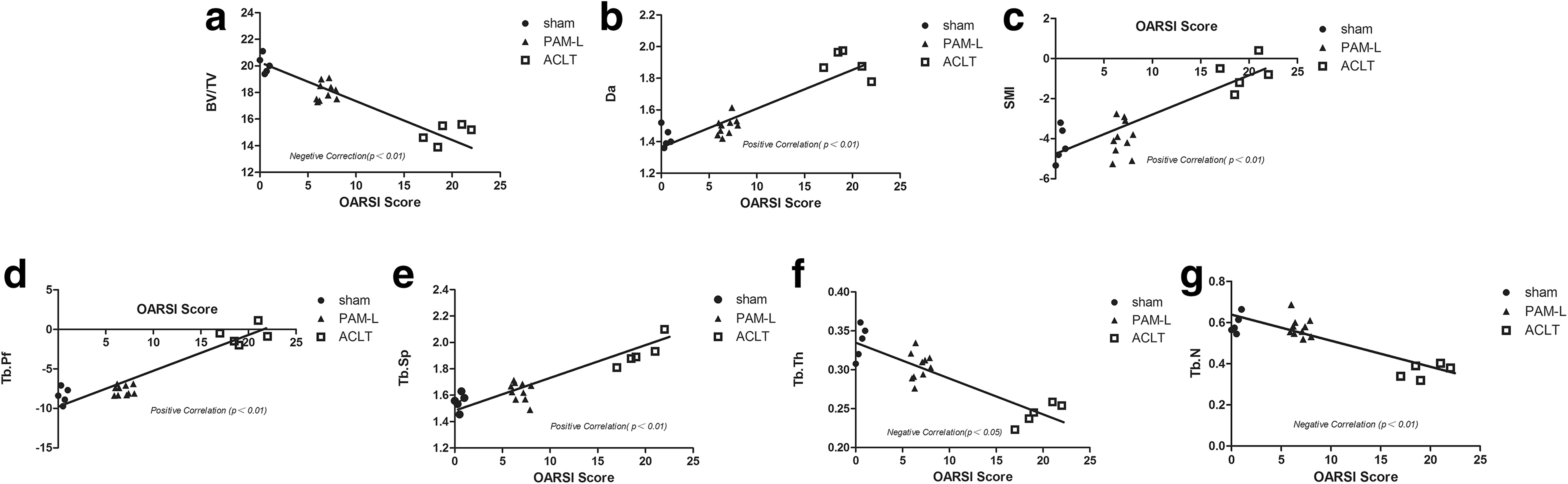

Supplement: Supplementary file 7 — Authors’ original file for figure 6 [file 12891_2014_2308_MOESM7_ESM.tiff]

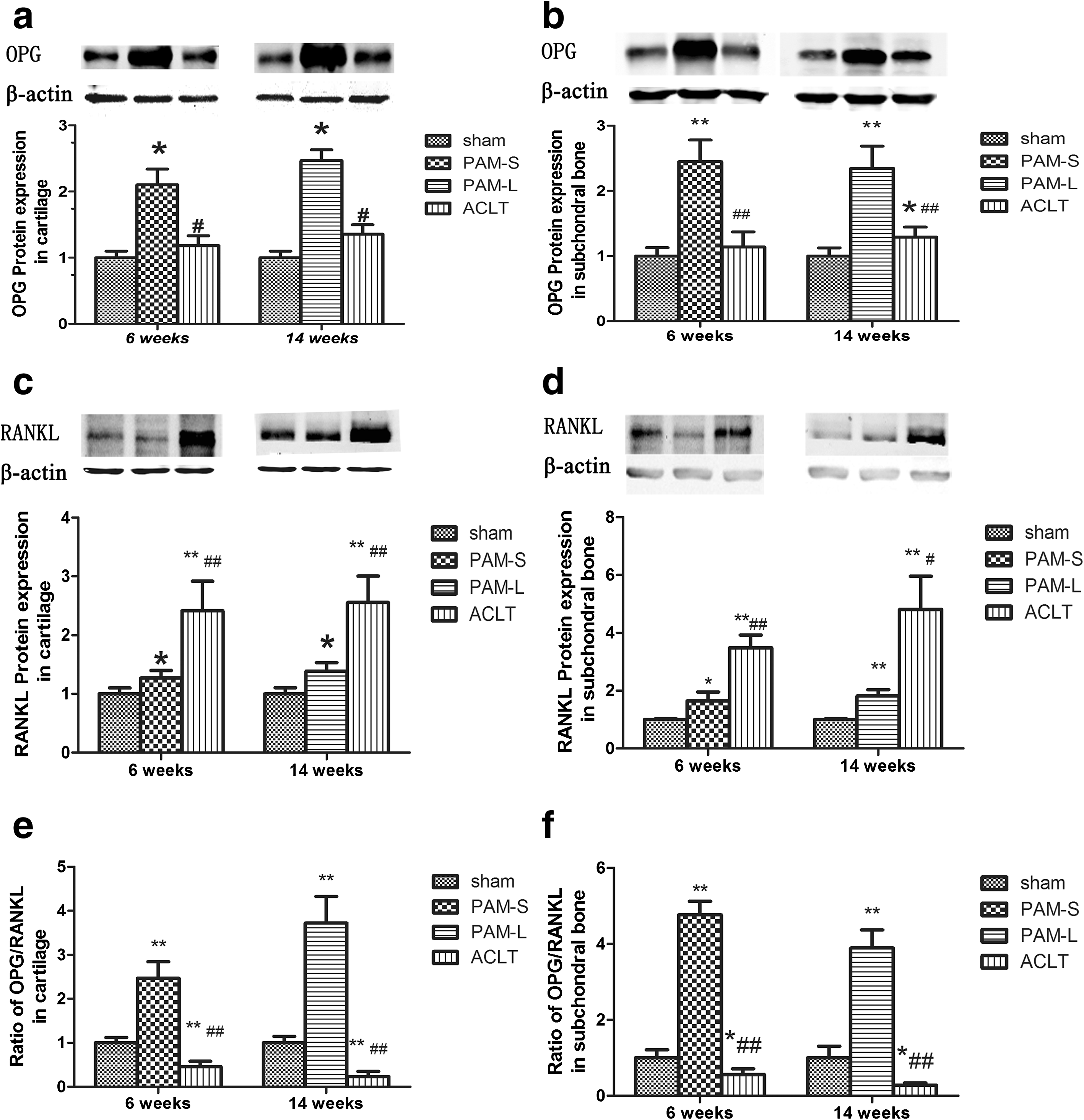

Supplement: Supplementary file 8 — Authors’ original file for figure 7 [file 12891_2014_2308_MOESM8_ESM.tiff]

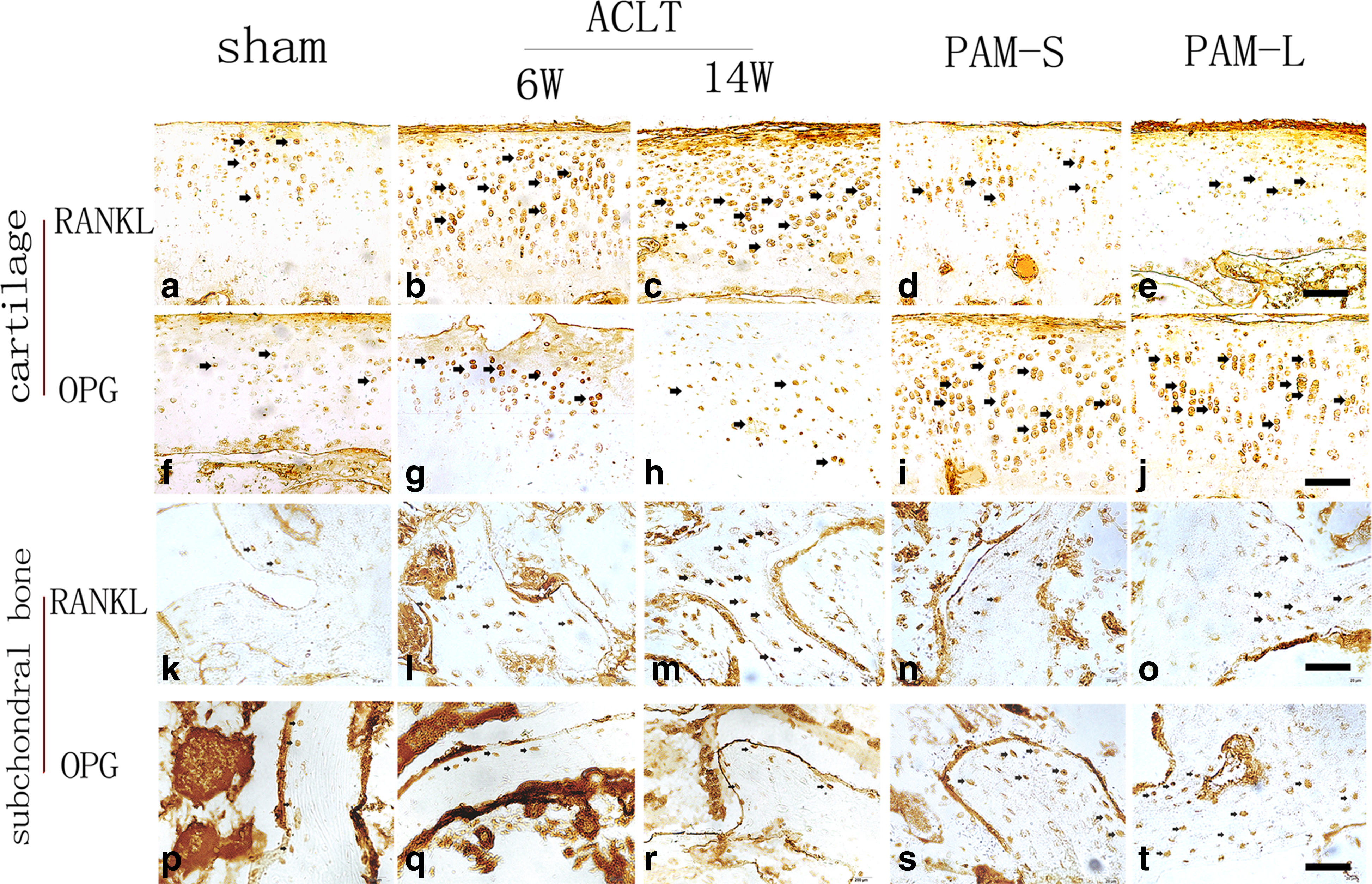

Supplement: Supplementary file 9 — Authors’ original file for figure 8 [file 12891_2014_2308_MOESM9_ESM.tiff]

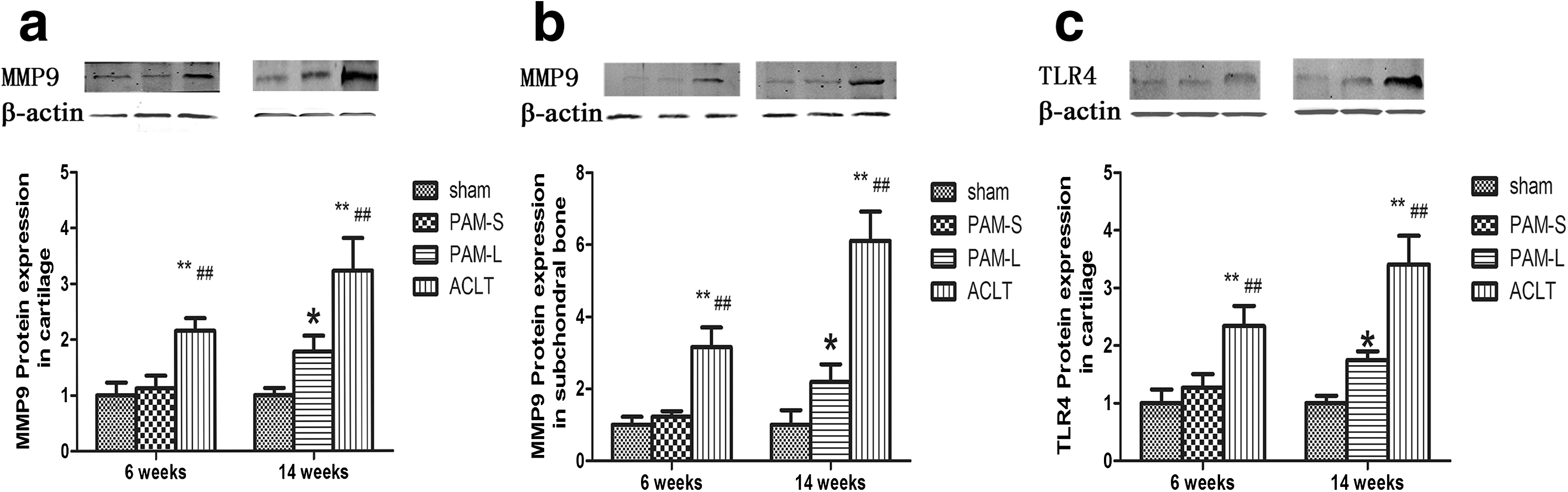

Supplement: Supplementary file 10 — Authors’ original file for figure 9 [file 12891_2014_2308_MOESM10_ESM.tiff]
